# Variable selection for disease progression models: methods for oncogenetic trees and application to cancer and HIV

**DOI:** 10.1186/s12859-017-1762-1

**Published:** 2017-08-01

**Authors:** Katrin Hainke, Sebastian Szugat, Roland Fried, Jörg Rahnenführer

**Affiliations:** Department of Statistics, TU Dortmund University, Dortmund, 44221 Germany

**Keywords:** Disease progression model, Oncogenetic tree, Variable selection

## Abstract

**Background:**

Disease progression models are important for understanding the critical steps during the development of diseases. The models are imbedded in a statistical framework to deal with random variations due to biology and the sampling process when observing only a finite population. Conditional probabilities are used to describe dependencies between events that characterise the critical steps in the disease process.

Many different model classes have been proposed in the literature, from simple path models to complex Bayesian networks. A popular and easy to understand but yet flexible model class are oncogenetic trees. These have been applied to describe the accumulation of genetic aberrations in cancer and HIV data. However, the number of potentially relevant aberrations is often by far larger than the maximal number of events that can be used for reliably estimating the progression models. Still, there are only a few approaches to variable selection, which have not yet been investigated in detail.

**Results:**

We fill this gap and propose specifically for oncogenetic trees ten variable selection methods, some of these being completely new. We compare them in an extensive simulation study and on real data from cancer and HIV. It turns out that the preselection of events by clique identification algorithms performs best. Here, events are selected if they belong to the largest or the maximum weight subgraph in which all pairs of vertices are connected.

**Conclusions:**

The variable selection method of identifying cliques finds both the important frequent events and those related to disease pathways.

**Electronic supplementary material:**

The online version of this article (doi:10.1186/s12859-017-1762-1) contains supplementary material, which is available to authorized users.

## Background

Disease progression models describe the step-wise development of diseases over time. The steps are defined by binary events that occur at different stages of the disease. A disease progression model represents the dependencies between these events, mostly by specifying assumptions on the order and the independence of pairs of events. The goal of these models is a better understanding of disease progression and in the long run support for medical decision making in terms of dose selection and therapy choice, based on individual disease trajectories.

In the literature, many explicit probabilistic model classes have been proposed and analysed, starting with a simple path model [1]. The list of extensions includes oncogenetic trees [2], distance based trees [3], directed acyclic graphs [4], contingency trees [5], oncogenetic tree mixture models [6], network aberration models [7], conjunctive Bayesian networks and their extensions [8–10], hidden-variable oncogenetic trees [11], progression networks [12] as well as new techniques to infer probabilistic progression like RESIC [13, 14], CAPRESE [15] and CAPRI [16].

Hainke et al. [17] compare several progression model classes and discuss their advantages and disadvantages. In simulation studies data are drawn from predefined models and the ability to recapture the true model is examined. In this analysis the number of events is always fixed. However, often not all events that have been measured or that are available for model building should be included in the final model. This is especially relevant for modern high-dimensional genetic data. Variable selection for disease progression models has not been analysed in detail in the literature. Here, we present a comprehensive analysis of variable selection methods for oncogenetic trees. We introduce ten different methods to identify the important events of disease progression. By means of a simulation study, we compare these methods for several data situations. We choose the oncogenetic trees for our analysis, because they are a very simple but popular, easy to understand and yet flexible model class.

The events that are the basis for our disease progression models are typically clinicopathological and genetic measurements. In this paper, as practical examples we consider glioblastoma and meningioma, two brain tumour types, where the events are chromosomal aberrations in the tumour tissue, and HIV, where the events are mutations in the viral genome. We apply our variable selection methods to these data sets and compare the selected events and the corresponding tree models to the ones found in the literature.

## Methods

### Oncogenetic trees

Oncogenetic trees [2] describe disease progression by the ordered accumulation of genetic events. In many applications the genetic events are chromosomal aberrations, i.e. gains and losses on chromosome arms, which are assumed to be non-reversible, but all other events that can be described by binary variables could also be used. An oncogenetic tree is a directed tree whose vertices represent genetic events and whose edges represent transitions between these events. Each edge is weighted with the conditional probability of the child event given that the parent event has already occurred.

Formally, an oncogenetic tree *T*=(*V, E, r*,*α*) is defined by a set *V* of vertices (genetic events), a set *E* of edges (relationship between events), the root vertex *r* (starting point of the disease) and a map *α*:*E*→[0,1] (conditional probabilities) such that:

- (*V,E*) is a branching, that means each vertex has at most one incoming edge.
- The vertex *r* is the null event and has no incoming edge.
- There are no cycles.
- For all edges *e*=(*i,j*)∈*E*,
  - *α*(*e*)=*P*(*j*=1|*i*=1) is the conditional probability of event *j* given event *i* has already occurred,
  - *α*(*e*)>0 (if *α*(*e*)=0, we can delete *e* from *E*),
  - *α*(*e*)<1 if *e*=(*r,i*), i.e. *e* leaves the root (otherwise merge *r* and *i*).

One can characterise a probability distribution over the power set 2^*V*^ and calculate the probability that every event in *S*⊆*V* is observed in the following way. If *r*∈*S* and *E* ^′^⊆*E* such that *S* contains all vertices reachable from *r* in the tree *T* ^′^=(*V,E* ^′^,*r*,*α*), then

$$p(S) = \prod_{e \in E'}{\alpha(e)} \cdot \prod_{\substack{e=(u,v) \in E \\ u \in S, v \notin S} }(1-\alpha(e)). $$

If *E* ^′^ is empty for the constraints mentioned above, then *p*(*S*)=0. Thus, some sets of genetic events have probability 0 and are not represented by the tree.

To specify the tree structure, one defines edge weights *w*_*ij*_ for every combination of events based on relative frequencies estimated from the data:

$$\begin{array}{*{20}l} w_{ij} & = \text{log}\left(\frac{p_{i}}{p_{i} + p_{j}} \cdot \frac{p_{ij}}{p_{i} p_{j}}\right) \\ & = \log(p_{ij}) - \log(p_{i} + p_{j}) - \log(p_{j}) \end{array} $$

with *p*_*i*_:=*P*(*X*_*i*_=1) and *p*_*ij*_:=*P*(*X*_*i*_=1,*X*_*j*_=1). Then, Edmonds’ branching algorithm [18] is used to find the rooted tree with maximum weight.

**Fig. 1:**
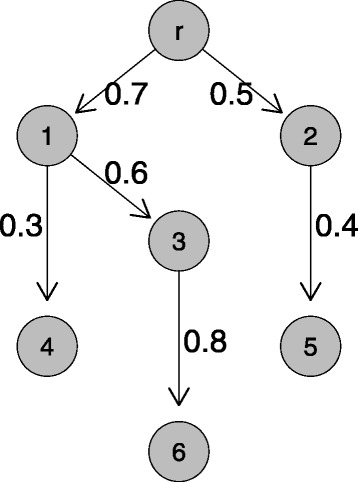
Example of an oncogenetic tree model with *n*=6 events. The edge weights represent the conditional probability that the child event occurs given that the parent event already occurred

An example of an oncogenetic tree model with *n*=6 events is given in Fig. 1.

### Variable selection methods

In this section we introduce ten variable selection methods. The goal is to separate the events that are relevant for disease progression from those representing only random noise. Starting point for the variable selection is a binary data matrix $X = \left [ \mathbf {x_{1}}, \dots, \mathbf {x_{n}} \right ] \in \mathbb {B}^{m \times n}$ that represents the occurrence of *n* genetic events in *m* observations, i.e. *x*_*i*_ is a vector of length *m* corresponding to the genetic event *i*. The overall procedure then is to first identify the relevant subset of events and then fit an oncogenetic tree model using only the selected events.

Table 1 contains an overview of all variable selection methods considered here. The methods are divided into four groups. Two methods are based on univariate frequencies of events, three on pairwise interactions, three select events with benefit for the subsequently fitted oncogenetic tree, and two are based on the identification of cliques of events.

**Table 1:** Overview of all variable selection methods considered here

Name	Short name	Short description of criterion for selected events
Univariate Frequency	freq	Frequency above cutoff
Method of Brodeur	brod	High frequency, compared to uniform distribution
Pairwise Correlation	cor	Event pairs with high correlation
Fisher’s Exact Test	fisher	Event pairs with significant dependence
Fisher’s z-transformation	z	Event pairs with significant dependence
Weights of Edmonds’ Algorithm	weight	Event pairs with large weights in algorithm
Conditional Probabilites in Tree	OT	Large conditional probabilities in oncogenetic tree
Independence in Tree	single	Remove single independent events in fitted tree
Largest Clique Identification	lcliq	Member of the largest subgraph
Maximal Clique Identification	mcliq	Member of the maximum weight subgraph

Only two of these methods have been applied in the literature so far: the frequency based method freq [19–22] and the method of Brodeur brod [4, 23–30]. We add and investigate some new proposals based on the following concepts. Since oncogenetic trees represent dependencies between events, one idea is to consider this by means of pairwise correlation or pairwise independence. Another approach is to use some main aspects of the underlying tree fitting algorithm. This includes the weights used in the construction algorithm, the conditional probabilities in the resulting tree as well as the tree representation of independent events.

#### Univariate frequency

A simple intuitive approach is to select all events with a relative frequency of occurrence in the underlying data set above a fixed threshold *τ*_**freq**_∈(0,1). An event *i*∈{1,…,*n*} is selected if $\overline {x_{i}} \ge $ *τ*_**freq**_, with $\overline {x_{i}} = \frac {1}{m} \sum _{k=1}^{m}{x_{i}^{k}}$ where $x_{i}^{k}$ is the *k*-th component of *x*_*i*_.

#### Method of Brodeur

Brodeur et al. [23] proposed a method to identify nonrandom events in human cancer data. Under the null hypothesis that all events occur randomly, they assume that the events occur independently and with equal probability. Using this uniform prior, one can compare the distribution of observed and expected events. By means of a Monte Carlo simulation one generates 10 000 random data sets to obtain the frequencies for each event under the null hypothesis. For each of the 10 000 replicates the maximum frequency is recorded. Then an event is considered nonrandom, if the observed frequency exceeds the 95th percentile of these maximum scores, i.e. $\overline {x_{i}} \ge \tau _{\mathbf {freq}}^{*}$, where $\tau _{\mathbf {freq}}^{*}$ is the mentioned 95th percentile.

The method of Brodeur is a frequency-based selection procedure, where the threshold is not defined in advance, but is calculated by the selection procedure itself.

If one uses data sets where the events are mutations on chromosome arms, Brodeur et al. suggest not to use the uniform distribution but a distribution taking the length of the chromosome arms into account. Using this length proportional null distribution one needs to calculate normalised frequencies for each event and to compare these to the normalised observed frequencies, see [23] or [26] for details.

#### Pairwise correlation

The idea of this method is to select all events with sufficient correlation to at least one other event. For binary events, Pearson’s correlation coefficient is equivalent to the phi coefficient. The pairwise correlation between events *i* and *j* (*i,j*∈{1,…*n*}) is defined by

$$r_{ij} := \frac{\sum_{k=1}^{m}{\left(x_{i}^{k} - \overline{x_{i}}\right)\left(x_{j}^{k} - \overline{x_{j}}\right)}}{\sqrt{\sum_{k=1}^{m}{\left(x_{i}^{k} - \overline{x_{i}}\right)^{2}} \sum_{k=1}^{m}{\left(x_{j}^{k} - \overline{x_{j}}\right)^{2}}}}, $$

where $x_{i}^{k}$ and $x_{j}^{k}$ are the *k*-th component of the corresponding vectors.

The definition of the phi coefficient that describes the association of event i and j is

$$\phi = \frac{n_{11}n_{00} - n_{10}n_{01}}{\sqrt{n_{1\cdot}n_{0\cdot}n_{\cdot 1}n_{\cdot 0}}}, $$

where *n*_11_ is the number of samples with events *i* and *j*, *n*_10_ the number of samples only with event *i*, and so on. Given the threshold *τ*_**cor**_∈(0,1) for the correlation, we select an event *i* if ∃ *j*∈{1,…,*n*}∖{*i*}:|*r*_*ij*_|≥*τ*_**cor**_.

#### Fisher’s exact test

Another approach based on interaction analysis is to apply Fisher’s exact test for pairwise independence [31]. We compute all $\binom {n}{2}$ p-values *p*_*ij*_ of event pairs (*i*, *j*) (*i,j*=1,…,*n*, *i*<*j*) and select all event pairs whose corresponding p-values indicate dependence. For a threshold *τ*_**fisher**_∈(0,1) we select both events *i* and *j* if *p*_*ij*_≤*τ*_**fisher**_.

#### Fisher’s z-transformation

A variable selection method also based on a test procedure uses confidence intervals for Pearson’s correlation coefficient. Pigott [32] suggests to first apply Fisher’s z-transformation to the correlation coefficient of event pairs to obtain an approximately normally distributed random variable. The transformation is defined as

$$z_{ij} = 0.5 \ln \left(\frac{1+r_{ij}}{1-r_{ij}} \right). $$

The asymptotic variance of *z*_*ij*_ is given by $\text {Var}(z_{ij}) = \frac {1}{m-3}$ such that

$$\text{CI} = \left[ z_{ij} - u_{1-\frac{\alpha}{2}} \cdot \frac{1}{\sqrt{m-3}}, z_{ij} + u_{1-\frac{\alpha}{2}} \cdot \frac{1}{\sqrt{m-3}} \right] $$

is an asymptotic (1−*α*) confidence interval, where $u_{1-\frac {\alpha }{2}}$ is the $(1-\frac {\alpha }{2})$ quantile of the standard normal distribution.

This confidence interval can be used for variable selection. We calculate all pairwise correlation coefficients *r*_*ij*_. If the corresponding confidence interval does not include 0 (0∉ CI),

we select both events *i* and *j*. The threshold in this case is defined by ***τ***_**z**_=1−*α*∈(0,1).

#### Weights *w*_*ij*_ of Edmonds’ branching algorithm

Another approach is to use the weights of Edmonds’ branching algorithm that are the basis for the construction of an oncogenetic tree. Only those events are selected that are associated with large weights *w*_*ij*_, defined by

$$w_{ij} = \text{log}\left(\frac{p_{i}}{p_{i} + p_{j}} \cdot \frac{p_{ij}}{p_{i} p_{j}}\right) $$

for *i,j*=1,…,*n, i*≠*j*. We first determine the maximum of *w*_*ij*_ and *w*_*ji*_, since a fitted tree would rather contain the edge with the larger weight. Let this be w.l.o.g. *w*_*ij*_. Then we set a relative threshold *τ*_**weight**_ ∈(0,1) and determine the ⌈100·*τ*_**weight**_⌉*%* largest weights *w*_*ij*_. All events corresponding to at least one of these weights are then selected.

#### Conditional probabilities in tree

In contrast to all variable selection methods presented so far, we now fit an oncogenetic tree *T*=(*V, E, r*,*α*) to the entire data set with *n* events. Then we select those events whose adjacent edges have sufficiently large conditional probabilities i.e. edge weights. All edges (*i,j*),(*j,k*)∈*E* are called adjacent to event *j*. Let *τ*_**OT**_∈(0,1) be the minimally required conditional probability. We include event *j* in our final model if

$$\max(\alpha(e), \alpha(f): e = (i,j) \in E, f = (j,k) \in E) \ge \boldsymbol{\tau}_{\mathbf{OT}}. $$

Note that *e* is clearly defined since all vertices in the tree except *r* have exactly one parent, whereas there can be more than one edge *f*, because each vertex can have several children.

#### Independence in tree

We again fit an oncogenetic tree to the entire data set. Events that are independent from all others are represented as vertices directly leaving the root with no children. We remove these independent events. The remaining events represent our set of selected variables.

Note that this kind of variable selection method does not imply that independent events are always unnecessary or not important for disease progression.

#### Clique identification

The last two methods are based on the identification of cliques. A clique *C* is a subgraph of an undirected graph *G*_*u*_=(*V, E, w*), with *w* being the edge weights, where all pairs of vertices are connected by an edge. The idea to determine a clique with certain properties as a variable selection method originates from Desper et al. [2].

As a start, consider the complete graph $G_{c} = (V, \tilde E, w)$, where all *n* events are pairwise connected, i.e. $\tilde E = \{e = (i,j): i,j \in 1, \dots, n, i < j\}$. As edge weights *w* we use the weights *w*_*ij*_ of Edmonds’ branching algorithm. Thus define $w: E \rightarrow \mathbb {R}_{+}$ with *w*(*e*)=*w*_*ij*_+*w*_*ji*_, *e*=(*i,j*). Using the sum of these edge weights we include both directions in the undirected graph. To enable the clique identification we delete edges from *G*_*c*_ and obtain *G*_*u*_. Desper et al. delete those edges *e*=(*i,j*) whose vertices *i* and *j* have not been observed simultaneously at least five times in the data set.

For our variable selection method we define a relative frequency *τ*_**clique**_∈(0,1) instead of an absolute one as suggested by Desper et al. and delete an edge *e*=(*i,j*) from *G*_*c*_ if

$$\frac{1}{m} \sum_{k=1}^{m}{I\left(\left(x_{i}^{k} = 1\right) \wedge \left(x_{j}^{k} = 1\right)\right)} < \boldsymbol{\tau_{\mathbf{clique}}}, $$

where *I* is the indicator function. Let *F* denote the set of deleted edges, then $E = \tilde E \backslash F$ is the resulting set of edges in the undirected graph *G*_*u*_.

Starting from *G*_*u*_ we present two variable selection methods: lcliq is based on the largest clique and mcliq on the maximal clique. An illustrating example concerning the difference between largest and maximal cliques is given in Additional file 1: Figure A.1.

A clique *C* is called largest if there is no other clique including more vertices. The events of this largest clique are chosen for the final model fit. It is possible that *C* is not unique. There might be more than one clique with the same largest number of vertices. In this case we select all events from all largest cliques.

A clique *C* is called maximal if it cannot be extended to a larger clique. The largest cliques are always maximal, but a maximal clique is not necessarily largest. We identify all maximal cliques *C*_1_,…,*C*_*q*_ of *G*_*u*_, *C*_*i*_=(*V*_*i*_,*E*_*i*_,*w*). The maximum-weight clique then is

$$C:= \arg \max_{C_{i}} \sum_{e \in E_{i}}{w(e)}. $$

The set *V*_*i*_ of vertices of this maximal clique with maximum weight represents the selected subset of events.

## Results

### Comparison of variable selection methods by means of a simulation study

In this section we evaluate the ten variable selection methods presented above. First, we describe the design of the simulation study. Then, we choose a suitable threshold separately for each variable selection method. And finally, using these best threshold values, we compare all methods and identify the best one(s).

#### Design of the simulation study

The following evaluation procedure is used to evaluate the ten variable selection methods, see also the detailed explanation afterwards.

1. Sample a random oncogenetic tree *T* with *n*_1_ events.
2. Sample *m* observations from *T* and obtain a data matrix $X \in \mathbb {B}^{m \times n_{1}}$.
3. Sample *m* observations from *Y*_*i*_∼Bin(1,*π*_*i*_), with *π*_*i*_∈(0,1), *i*=1,…,*n*_2_.
4. Combine the data from step (2) and (3) to a data matrix $\tilde X \in \mathbb {B}^{m \times (n_{1} + n_{2})}$.
5. Apply a variable selection method to $\tilde X$ and obtain a data matrix *X* ^∗^ containing only the selected events.
6. Fit an oncogenetic tree *T* ^∗^ to *X* ^∗^.
7. Compare *T* ^∗^ to *T*.
8. Compare *X* ^∗^ to *X*.

The oncogenetic tree *T* is the underlying true model. This tree is generated randomly in step (1), with a fixed number *n*_1_ of events and a fixed interval [*α*_*l*_,*α*_*u*_] (0<*α*_*l*_<*α*_*u*_<1) for the edge weights. Here, the Prüfer encoding of trees is used to draw a tree uniformly at random from the tree topology space [33, 34]. In a next step, we generate a random data matrix $X = \left [ \mathbf {x_{1}}, \dots, \mathbf {x_{n_{1}}} \right ]$ with *m* observations from *T*. (We do not simulate waiting and sampling times.) Ideally, these *n*_1_ events would in the end be reidentified by our variable selection methods. To make the selection process more difficult and realistic, we draw realizations from a binomial random variable with parameter *π*_*i*_ for *n*_2_ further events, see step (3). We call these *n*_2_ additional events ’noise events’, because not every observable event is associated with the disease process, some are just random mutations. Note that this definition of noise events should not be mixed up with independent white noise that is used to represent uncertainty in the data generating process. We do not simulate measurement errors in our data, so far. Next we join the true and noise events to a single data matrix $\tilde X \in \mathbb {B}^{m \times (n_{1} + n_{2})}$. Then, in step (5), we apply a variable selection method to this data matrix. Each method selects *p*≤*n*_1_+*n*_2_ columns from $\tilde X$. This choice is denoted by *X* ^∗^ and one can fit an oncogenetic tree *T* ^∗^ to this data set.

To evaluate the performance of the selection methods, we compare the true and the fitted tree, *T* and *T* ^∗^, and also the true and the selected events, i.e. the data matrices *X* and *X* ^∗^.

The comparison of different tree models can be based on the induced probability distribution [17]. Assume we have two oncogenetic trees *T*_1_ and *T*_2_, each with *n* events. The two probability vectors for the 2^*n*^ combinations of events are denoted by *p*_1_ and $\mathbf {p_{2}} \in [0,1]^{2^{n}}$. Distances between these two vectors, i.e. between the two tree models, can then be calculated by the *L*_1_-distance, *L*_2_-distance and cosine-distance:

$$\begin{aligned} d_{L_{1}}(\mathbf{p_{1}}, \mathbf{p_{2}}) & = \sum_{i=1}^{2^{n}}{|p_{1_{i}} - p_{2_{i}}|}, \\ d_{L_{2}}(\mathbf{p_{1}}, \mathbf{p_{2}}) & = \sqrt{\sum_{i=1}^{2^{n}}{(p_{1_{i}} - p_{2_{i}})^{2}}} \\ d_{cos}(\mathbf{p_{1}}, \mathbf{p_{2}}) & = 1 - \cos \varangle(\mathbf{p_{1}},\mathbf{p_{2}}) = 1 - \frac{<\mathbf{p_{1}},\mathbf{p_{2}}>}{||\mathbf{p_{1}}|| \ ||\mathbf{p_{2}}||} \\ & = 1 - \frac{\sum_{i=1}^{2^{n}}{p_{1_{i}} \cdot p_{2_{i}}}}{\sqrt{\left(\sum_{i=1}^{2^{n}}{p_{1_{i}}^{2}}\right) \cdot \left(\sum_{i=1}^{2^{n}}{p_{2_{i}}^{2}}\right)}} \end{aligned} $$

The cosine-distance denotes the angle spanned by the two probability vectors.

Applying these distance measures in our simulation study, notice that *T* and *T* ^∗^ may contain different events, because of the selection process. The number of events can also differ. Thus, we need to consider all *n*_1_+*n*_2_ events when calculating the induced probability distribution. Combinations of events which contain an event that is not present in the underlying tree are assigned probability 0. Thus, the Kullback-Leibler divergence [35] as a potential measure of discrepancy between probabilities is not applicable.

Another way to evaluate variable selection methods, step (8), is to examine the false positives and false negatives, i.e. count how many of the noise events have not been detected and how many of the true events have been removed. These absolute counts are converted to relative ones. In order to have two criteria whose best value is 1, we calculate the converse probability for the proportion of removed true events. Thus, the criteria sens (for sensitivity) and spec (for specificity) measure the proportion of correctly identified true events respectively correctly removed noise events.

In the evaluation procedure mentioned above, there are some parameters that need to be defined in advance. These are the number *n*_1_ of true events, the number *n*_2_ of noise events, the number *m* of observations, the interval [*α*_*l*_,*α*_*u*_] for the edge weights and the probability *π*_*i*_ for the proportion of noise.

Based on these parameters, one can investigate data situations with different degrees of difficulty for the variable selection methods. In this simulation study, we choose two different values for each parameter (parameter *π*_*i*_ is sampled randomly and independently from the given interval for each noise variable):

$$\begin{aligned}  n_{1} & \in  \{5, 7\}\\  n_{2} & \in \{2, 12\}\\  m & \in \{50, 1000\}\\  \left[{\alpha}_{l}, {\alpha}_{u}\right] & \in \{[0.2, 0.8], [0.5, 0.8]\}\\ \pi_{i} & \in \{I_{0.1} = [0, 0.2], I_{0.3} = [0.2, 0.4]\} \end{aligned} $$

The full factorial experiment with all 32 parameter combinations is given in Additional file 1, Table B.1. In the simulation study presented in the following, we focus on 8 of these 32 parameter settings, since it turned out that not every parameter has a relevant influence on the results. If we cluster the *L*_1_-distances (see Additional file 1: Figure A.2) those distances are the smallest, where only *n*_1_ differs and the other four parameters are fixed. The value of *n*_1_ does not influence the results strongly. The same holds for the lower probability *α*_*l*_ of edge weights. In 6 out of 8 times, the second closest distances refer to parameter combinations with differences only in *α*_*l*_. Thus, only *n*_1_=5 and *α*_*l*_=0.2 are considered in the following. Combining the remaining three variables *n*_2_,*m* and *π*_*i*_ leaves us with 8 different parameter settings.

In addition, we also need to identify a suitable threshold for each variable selection method. We choose four different values for each method. In further simulations smaller or higher values did yield worse results.

$$\begin{array}{*{20}l} \tau_{\text{freq}} & \in \{0.05, 0.10, 0.15, 0.20 \} \\ \tau_{\text{cor}} & \in \{0.10, 0.20, 0.30, 0.40 \} \\ \tau_{\text{fisher}} & \in \{0.01, 0.05, 0.10, 0.15 \} \\ \tau_{\text{z}} & \in \{0.50, 0.63, 0.77, 0.90 \} \\ \tau_{\text{weight}} & \in \{0.05, 0.10, 0.20, 0.30 \} \\ \tau_{\text{OT}} & \in \{0.10, 0.15, 0.20, 0.25 \} \\ \tau_{\text{lcliq}} & \in \{0.05, 0.10, 0.15, 0.20 \} \\ \tau_{\text{mcliq}} & \in \{0.05, 0.10, 0.15, 0.20 \} \end{array} $$

For each parameter combination we generate *M*=100 random oncogenetic trees with corresponding data sets. We apply ten different variable selection methods, each with four different thresholds (except the method of Brodeur where the threshold is calculated implicitly and the method of independence in trees with no threshold at all). Based on these results, we evaluate our methods.

All variable selection methods as well as our evaluation procedure are implemented in the statistical programming language R, version 3.0.1 [36]. We used the R packages Rtreemix [37] to fit oncogenetic trees and igraph [38] to perform the clique calculations. The execution of all methods is computationally feasible.

#### Results: choosing the best threshold

We first determine a suitable threshold for each variable selection method. For this purpose, we focus on the *L*_1_-distance, because the results do not differ much for the *L*_2_- or cosine-distance, see Additional file 1: Figure A.3. Using the other two criteria sens and spec is not meaningful, since both criteria need to be considered simultaneously and this would always lead to contradictory thresholds. Concerning the criterion sens one would choose the highest threshold and concerning spec the lowest, or vice versa.

**Fig. 2:**
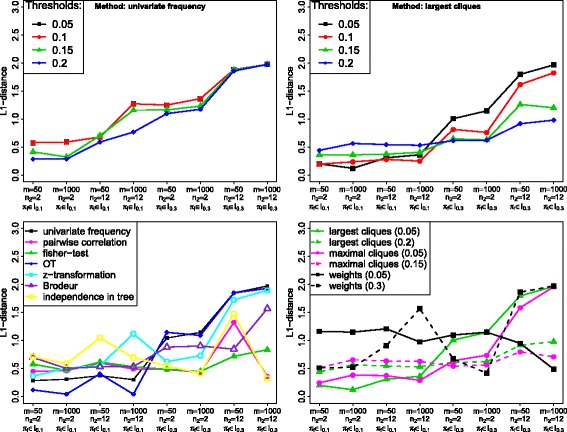
Results of the simulation study. The eight different parameter settings are displayed on the x-axis whereas the means of the 100 L _1_-distances for combinations of method and threshold are shown on the y-axis. *Top left:* Results for the univariate frequency method with all chosen thresholds. *Top right:* Results for the largest cliques method with all chosen thresholds. *Bottom left:* Comparison of seven different selection methods, each with one threshold that was globally best for all parameter situations. *Bottom right:* Comparison of three different selection methods. The chosen threshold is given in brackets, because there was no globally best one

Using the *L*_1_-distance, the results for the univariate frequency method freq are shown in Fig. 2 (top left). On the x-axis, one can see the 8 different parameter settings. The y-axis shows the mean of the 100 *L*_1_-distances between the fitted model and the true model. The four different lines represent the four different thresholds.

One can see that for the first four parameter settings with proportion of noise *π*_*i*_∈*I*_0.1_=[0,0.2] the distances are smaller than for *π*_*i*_∈*I*_0.3_=[0.2,0.4], where the highest considered threshold is *τ*_freq_=0.2. In this case *τ*_freq_ is clearly below the proportion of noise such that noise events are not eliminated in the variable selection step.

Choosing *τ*_freq_=0.2 leads to the best or nearly best results for all parameter settings. An even larger threshold would improve the results for *π*_*i*_∈*I*_0.3_, but is unrealistic for most applications we have in mind.

Figure 2 (top right) displays the results for the largest cliques method lcliq. Again, we observe larger distances to the true model for higher proportion of noise events. In data situations with low proportion of noise events (*π*_*i*_∈*I*_0.1_), the order from best to worst threshold (in terms of the smallest *L*_1_-distances) is from the lowest to the highest value. For a high noise proportion (*π*_*i*_∈*I*_0.3_), we discover exactly the opposite. Now, the highest threshold leads to the best result, whereas the lowest threshold performs worst. Thus, we need to adapt the threshold to the noise proportion.

The results for the other six methods are shown in Additional file 1: Figure A.4. In summary, Table 2 shows our recommendation, which threshold to use in which data situation.

**Table 2:** Recommendation of the thresholds to be used for each method and each data situation

*n*_1_	50/1000	50/1000	50/1000	50/1000
*n*_2_	2	12	2	12
*π*_*i*_	[0,0.2]	[0,0.2]	[0.2,0.4]	[0.2,0.4]
freq	0.2	0.2	0.2	0.2
cor	0.3	0.3	0.3	0.3
fisher	0.01	0.01	0.01	0.01
z	0.9	0.9	0.9	0.9
weight	0.3	0.05	0.3	0.05
OT	0.25	0.25	0.25	0.25
lcliq	0.05	0.05	0.2	0.2
mcliq	0.05	0.05	0.15	0.15

The method of Brodeur generates its threshold implicitly and the single method does not need any threshold at all

Note that the method of Brodeur brod requires no threshold choice, as it is part of the method. The mean thresholds for the 8 different data situations (and in brackets their standard deviations) are 0.38 (0.088), 0.26 (0.086), 0.30 (0.041), 0.19 (0.036), 0.46 (0.082), 0.33 (0.085), 0.49 (0.034), and 0.34 (0.035). Thus, they are almost always higher than the one we chose for the univariate frequency selection.

#### Results: comparison of variable selection methods via the *L*_1_-distance

Now, we compare the different variable selection methods. For this comparison, we choose the best thresholds from above. For the reason of clarity we first compare the seven selection methods with an overall best threshold separately from the other three methods with a situation-dependent threshold (see bottom of Fig. 2). The mean standard error for the data in these two figures is 0.034.

In the bottom left of Fig. 2, one can see that the z-transformation method z is never the best method. The correlation method cor as well as the independence in tree method single are among the best ones in two data situations (directly followed by the Fisher-test), but a lot worse in others. Thus z, cor and single are not considered any further. For noise proportion *π*_*i*_∈*I*_0.1_ the best methods are the oncogenetic trees OT and in one scenario the frequency method freq, whereas for higher noise values (*π*_*i*_∈*I*_0.3_) one should choose the Fisher-test fisher.

Figure 2 (bottom right) shows that in the case of little noise (*π*_*i*_∈*I*_0.1_) both clique methods lcliq and mcliq perform best (each with the lower threshold). If there is more noise *π*_*i*_∈*I*_0.3_) the method using the weights of Edmonds’ branching algorithm weight leads to the smallest *L*_1_-distances in two situations. However, one needs to know the number of noise variables in advance to choose the best possible threshold. Neglecting this weight method, the two clique methods are again the best, this time each one with the higher threshold.

**Fig. 3:**
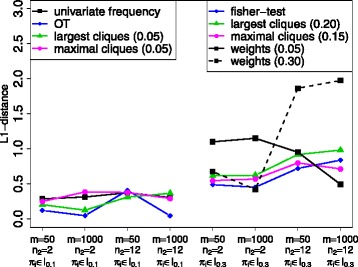
Comparison of all variable selection methods. Based on the results from Fig. 2 we need to distinguish between situations with low and high proportion of noise variables (*π*_*i*_∈*I*_0.1_ vs. *π*_*i*_∈*I*_0.3_)

Now, we summarise these results in Fig. 3 to find an overall best variable selection procedure. Based on the results shown in Fig. 2, we first compare the best methods subject to the amount of underlying noise. For *π*_*i*_∈*I*_0.1_ the best methods are the largest cliques lcliq and OT. However, having few observations and many noise variables OT performs worst. Thus, we propose to use the largest clique method with threshold 0.05. In the case of *π*_*i*_∈*I*_0.3_, fisher and mcliq (with threshold 0.15) perform best.

All in all, the clique methods show the globally best performance. They do not always achieve the best results, but they provide very good results for all data situations considered here, which no other method does. The largest cliques lcliq perform a little better in case of little noise and the maximal cliques mcliq in case of higher noise, but they do not differ substantially. In addition, one needs to select a suitable threshold. We propose to adaptively choose the low threshold for a low proportion of noise and the high threshold for a higher proportion of noise.

#### Results: comparison of variable selection methods via false positives and negatives

We now want to compare the performance of the variable selection methods with regard to the two criteria sens and spec. A good method should obtain high values for both criteria simultaneously, i.e. the method identifies most or all true events and removes most or all noise events. A method that is only good in one of these aspects is not convenient, since one can always achieve the best value for sens by selecting all events and the best value for spec by selecting no event.

The analysis of these false positives and negatives is performed analogously to the one of the *L*_1_-distance. For the reason of clarity we again compare the seven methods with one overall best threshold separately from the other three methods with a situation-dependent threshold. Afterwards we compare the best methods of each approach to identify the overall best method.

As a result, we discovered that in contrast to the *L*_1_-distance no separation between situations with *π*_*i*_∈*I*_0.1_ or *π*_*i*_∈*I*_0.3_ is necessary. But we also observed that the clique methods are not good in identifying the true events. Further investigations revealed that this is due to the parameter *α*_*l*_, which we set to the value 0.2, since it did not change the results for the *L*_1_-distance. It turned out that this is not true for the clique methods and the criterion sens. The explanation is that having a small value for *α*_*l*_ can lead to very low probabilities for the leaf-events. If a single event only occurs very seldom, e.g. less often than the clique threshold, it is impossible that this event is included in the selection process, since it cannot occur simultaneously with any other event sufficiently frequent.

**Fig. 4:**
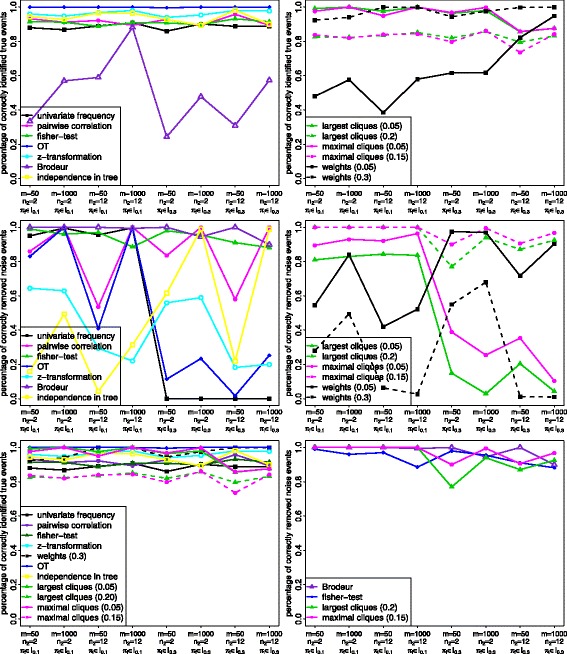
Results of the simulation study. The eight different parameter settings are displayed on the x-axis whereas the means of the 100 values for sens and spec are shown on the y-axis. For all figures it holds that *α*_*l*_=0.5 (instead of *α*_*l*_=0.2 for the *L*_1_-distance). *Top row:* Results for the criterion sens, *left*: comparison of all seven methods with one overall best threshold, right: comparison of all three methods with two thresholds depending on the underlying data situation. *Middle row:* Results for the criterion spec, left: comparison of all seven methods with one overall best threshold, right: comparison of all three methods with two thresholds depending on the underlying data situation. *Bottom row:* Comparison of all variable selection methods for the two criteria sens (*left*) and spec (*right*)

Thus, we now show the results for the same 8 data situations as before but with the parameter *α*_*l*_ set to the value 0.5, see Fig. 4. The results with *α*_*l*_=0.2 are shown in Additional file 1: Figure A.5 so that one can check that the major differences only concern the clique methods. Another representation of these results for sens and spec are shown in ROC-curves in Additional file 1: Figures A.6 and A.7.

Concerning the criterion sens (top row), one can see that nearly all methods with one overall best threshold perform well regarding the identification of true events. Only the method of Brodeur shows poor results. Furthermore, all clique methods (the lower threshold better than the higher one) and the weight-method with threshold 0.3 show good results. In contrast, with respect to the criterion spec (middle row), the only two adequate methods with one overall best threshold are brod and fisher. In addition, the two clique methods with the higher threshold also perform well. Thus, the clique methods can again be recommended, since they can identify both the true and the noise events (bottom row). Clique identification with a high threshold allows to remove noise events. Using the lower threshold is favourable for identifying true events. All in all, the higher threshold is recommended. Nevertheless, one needs to bear in mind that we consider only situations where the true events have a sufficient probability of occurrence due to the parameter *α*_*l*_=0.5. The second best method is the Fisher test, which also achieves high values for both sens and spec simultaneously.

If one is in doubt, whether the assumption of *α*_*l*_=0.5 holds in an underlying data set, one can choose the fisher method, since this is the only one with results mostly over 80*%* for both criteria and all data situations if *α*_*l*_=0.2, see Additional file 1: Figure A.5. Having a low probability for noise events, i.e. *π*_*i*_∈*I*_0.1_, one can still rely on the clique methods with a low threshold to perform good.

### Application to real data

We now apply all variable selection methods to three different data sets and compare the corresponding resulting tree models with models provided in the literature for the application scenarios.

#### Meningioma

The meningioma data set with 661 observations and 9 events is taken from Urbschat et al. [39]. Events represent chromosomal gains or losses on chromosomes or chromosome arms in brain tumours. The genetic state of a tumour is characterised by the most frequent pattern of event combinations, as observed in a set of clones for each tumour. For fitting a tree model, Urbschat et al. chose 9 events based on the frequency selection freq with a threshold of 1.8*%*. Thus, all other possible events occur in less than 1.8*%* of the tumours.

On this data set we apply all variable selection methods with corresponding best thresholds from our simulation study. The results are shown in Table 3. The methods based on the Fisher test fisher, the z-transformation z and the independence in tree single select all events, whereas the two clique methods lcliq and mcliq (high threshold) select none at all. Many events are selected using the correlation method cor, the weight method (high threshold) and the OT approach. Only three events or even less are selected based on freq, the Brodeur method brod, weight and the clique procedures with low threshold. We can assume a low proportion for the noise, because only 9 events occur in more than 1.8*%* of the cases. Thus, our simulation suggests to use the clique methods with a low threshold. In this case only the events 14−, 22− and 1*p*− are selected.

**Table 3:** List of events (meningioma and HIV data set) respectively number of events (glioblastoma data set) that were chosen by our variable selection methods using the thresholds from the simulation study (*x* = event was selected)

Method	freq	brod	cor	fisher	z	weight	weight	OT	single	lcliq	lcliq	mcliq	mcliq
threshold	0.2	-	0.3	0.01	0.9	0.05	0.3	0.25	-	0.05	0.2	0.05	0.15
MENINGIOMA data set													
Chr14-			x	x	x	x	x	x	x	x		x	
Chr22-	x	x	x	x	x			x	x	x		x	
Chr1p-			x	x	x	x	x	x	x	x			
Chr6-			x	x	x		x	x	x				
Chr10-			x	x	x	x	x		x				
Chr18-			x	x	x		x	x	x				
Chr19-			x	x	x		x		x				
ChrY-				x	x			x	x				
ChrX-				x	x				x				
HIV data set													
215 F,Y	x	x	x	x	x		x	x	x	x	x	x	x
41 L	x		x	x	x	x	x	x	x	x	x		x
70 R	x	x	x	x	x		x	x	x	x		x	
67 N	x		x	x	x		x	x	x	x		x	
219 E,Q			x	x	x		x	x	x	x		x	
210 W			x	x	x	x	x	x	x				
GLIOBLASTOMA data set													
	23	29	73	99	102	89	102	85	131	22	10	22	11

The thresholds for the method of Brodeur are 0.1,0.33 and 0.17 respectively

Because of the low number of only 9 events we added 39 additional noise variables representing possible gains and losses on the other chromosomes. Since the proportion for these noise events in the real data is less than 1.8*%*, we set the event frequency for all simulated additional variables to 0.5*%* and randomly draw all additional data from a binomial distribution with *π*=0.005. Results for all variable selection procedures for this extended data set are shown in Additional file 1, Table B.2.

Interestingly only the frequency methods freq and brod and the clique methods lcliq and mcliq select none of the additional noise variables. All other methods select some or even many false positives. Additionally, the methods brod and weight select more of the true nonrandom variables.

Assuming that the 9 original variables are the ’true’ ones, one could also try to find the best threshold for each method that distinguishes best between the two groups. These thresholds, again with the number of selected noise variables, are also given in Table B.2. Again, only the frequency and the clique methods manage to clearly separate ’true’ and random events.

**Fig. 5:**
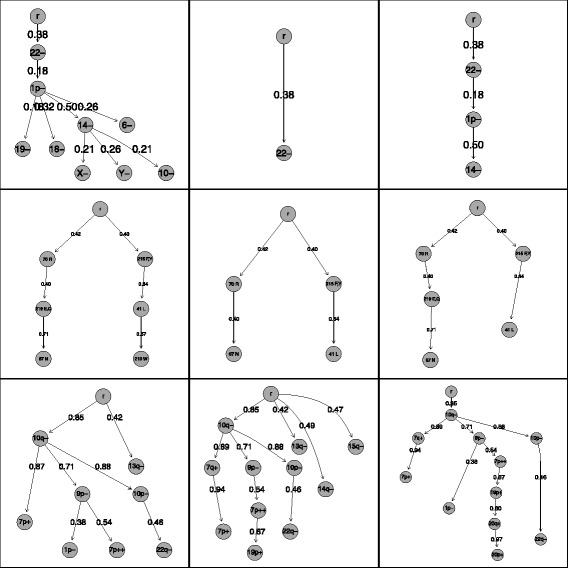
Some trees resulting from the variable selection process concerning the three data sets. The three rows represent the meningioma (*top*), HIV (*middle*) and glioblastoma (*bottom*) data sets, respectively. The columns show as a kind of reference tree the tree with all events (left), then two trees based on the frequency (*middle*) and clique selection (*right*)

We now compare the progression pathway of meningioma presented by [39] to the oncogenetic trees based on the results for the best variable selection methods freq and lcliq (largest clique), see Fig. 5, top row.

Precisely, we fit an oncogenetic tree to all events, resulting in a slightly different model as compared to [39] who fitted an oncogenetic tree mixture model instead of a single oncogenetic tree. We can see that even if the frequency and the largest clique method select very few events, they choose the important ones. Event 22− is the first event to occur and also the first event of all pathways. Thus, the occurrence of every other event depends on it. Although the frequency method selects only one event, it is at least the most important one. The largest cliques method lcliq selects three events and thereby covers the most frequent pathway. Thus, our variable selection methods detect the important events.

#### HIV

The HIV data set is also well studied [40], with knowledge about the existing pathways. This data set is available in the R package Rtreemix [37] and consists of 364 observations of 6 mutations that develop in the viral genome under zidovudine monotherapy. We again apply our ten variable selection methods to this data set. The results are also shown in Table 3.

Six out of thirteen selection methods (applying different thresholds) select all six events, whereas three methods select only two events. The frequency method freq and the two clique methods lcliq and mcliq (with low threshold) select four or five events. For the low number of only six events we assume a low proportion of noise and thus prefer the low threshold for the clique methods. We again show the comparison between the oncogenetic tree using all events and the ones based on the frequency and largest clique selection, see Fig. 5, middle row.

The tree with all six events shows two independent pathways with three variables each. Looking at the two other trees, one can recognise these two pathways as well. For the largest clique tree only event 210*W* at the end of one pathway is left out. This event is missing in the frequency tree as well. Interestingly, the second missing event by the frequency method is one in the middle of a pathway.

In summary, the results of many variable selection methods are quite similar here. We expected that many or all events will be selected, which was achieved by many methods including our promising clique techniques.

#### Glioblastoma

This glioblastoma data set is taken from the public data base ’The Cancer Genome Atlas’ and was preprocessed by Laura Tolosi [41, 42]. We have a binary data matrix with 539 observations of 132 events. The events are gain (+), loss (-) and amplification (++) of the chromosome arms 1 to 22.

The number of events in the glioblastoma data set is by far larger than in the meningioma and the HIV data set. Applying the variable selection methods using the thresholds from our simulation study often leads to a very large number of selected events, see Table 3. Again, the frequency and the clique methods yield the most reasonable results. They choose between 10 and 23 events, which are still manageable numbers for oncogenetic trees. All other methods select at least 73 events. This is not acceptable as estimated trees then become very unstable.

Thus, we decided to limit the number of selected events to approximately 11. We choose this number, because the maximal clique method mcliq (high threshold) selects 11 events and this method yields good results both in the simulation study and for the application to the other data sets. One should choose the high threshold here, because for 132 events in total we assume a higher proportion of noise events.

To select approximately 11 events, we choose the following thresholds for the selection methods: *τ*_freq_=0.41, *τ*_cor_=0.70, *τ*_fisher_=10^−26^, *τ*_weight_=0.0018, *τ*_OT_=0.90, *τ*_lcliq_=0.2, *τ*_mcliq_=0.15. We exclude the z-transformation, since even with a threshold of *τ*_z_=1−10^−16^ the method still selects 60 events and is therefore no reasonable choice. The method of Brodeur selects 29 events, based on the computed threshold $\tau _{\text {freq}}^{*} = 0.1725$. The independence in tree method single removes only one of the 132 events and is therefore useless on this data set and excluded from further comparison.

The results of the variable selection methods are given in Table 4. The frequency method freq selects almost all events mentioned in the literature [43], plus some additional ones. This is no surprise because the frequency method is a very common variable selection method. The method of Brodeur brod selects 29 events, including all events mentioned in the literature. The methods based on the pairwise correlation cor, the conditional probabilities of the oncogenetic tree OT and the weights of the branching algorithm weight detect only one or zero known events, whereas the Fisher method fisher identifies four out of eight known events. The clique methods lcliq and mcliq select almost all known events. Only the event 13q- (and for the largest clique also 1p- and 22q-) was not included in their selection.

**Table 4:** List of events from the glioblastoma data set that were chosen by our variable selection methods (*x* = event was selected)

Method	freq	brod	cor	fisher	weight	OT	lcliq	mcliq
threshold	0.41	0.1725	0.70	10^−26^	0.0018	0.90	0.2	0.15
**Chr7p+**	x	x	x	x		x	x	x
Chr7q+	x	x	x	x		x	x	x
Chr19p+	x	x	x	x			x	x
Chr20p+		x	x	x		x	x	x
Chr20q+		x	x	x		x	x	x
**Chr10p-**	x	x		x			x	x
**Chr10q-**	x	x		x			x	x
**Chr7p++**	x	x		x			x	x
**Chr9p-**	x	x					x	x
Chr19q+		x	x	x			x	
Chr9q++			x		x	x		
Chr12p++			x		x	x		
Chr18p++			x		x	x		
Chr18q++			x		x	x		
Chr21q++			x		x	x		
**Chr22q-**	x	x						x
**Chr1p-**		x						x
Chr2q++			x		x			
Chr3p++					x	x		
Chr8q++					x	x		
Chr11p-		x		x				
Chr11q-		x		x				
**Chr13q-**	x	x						
Chr14q-	x	x						
Chr15q-	x	x						
Chr1q+		x						
Chr1q-		x						
Chr3q-		x						
Chr4q-		x						
Chr6p-		x						
Chr6q-		x						
Chr8p-		x						
Chr9q-		x						
Chr12q+		x						
Chr12q-		x						
Chr15q+		x						
Chr21p-		x						
Chr7q-					x			
Chr13q+					x			
Chr18p+					x			

The events are sorted according to their selection frequency. Events already mentioned in the literature are printed in bold

Again, we compare the resulting trees for all selection methods. First, we look at the tree including only the events mentioned in the literature and compare it to the frequency and the maximal clique tree, see Fig. 5, bottom row. The literature tree is exactly included in the frequency tree, because the dependency structure does not change if we consider more events. Only the path 10*q*−→7*p*+ is slightly different, because the event 7*q*+ is inserted in the middle. Two other additional events are estimated as independent events directly leaving the root, and a third one is extending one pathway.

The tree resulting from the maximal clique method also contains the structure of the literature tree (again with the insertion of 7*q*+ in one pathway). Only the event 13*q*− is missing. This can be neglected, because this event is independent from all other pathways. The other additional events in this tree extend the existing pathway of 9*p*− and 7*q*++. Thus, the events 19*p*+, 20*p*+ and 20*q*+ might contain some further information concerning the progression of glioblastoma. In addition, these three events were selected by 6 of the 8 variable selection methods. Only two other events were selected more often. The frequency method, which can be considered as the standard method, detects only one of these three events. Thus, the maximal clique method mcliq is again convincing, as it identifies the important events already known in the literature and also some promising additional ones.

Looking at the trees resulting from the other selection methods (see Additional file 1: Figure A.8), we see that the Brodeur tree includes all pathways from the maximal clique tree, but also a lot more. Thus, it is difficult to identify the most important events and pathways. The correlation tree contains only two events from the literature, but also the new ’ 19*p*+→20*q*+→20*p*+’ pathway. The other six events are highly connected (edge weight 1), but occur almost never (edge weight 0.011). The Fisher test method performs only slightly worse than the maximal clique method, and the resulting tree contains the most important pathways. The weight method is useless, because the initial event occurs only in 3% of the samples. Some other events in the tree are highly correlated, but from this tree one cannot make any reasonable statement concerning progression in glioblastoma. The same holds for the OT tree. Two events from the literature are identified, but seven events are included in pathways with too small edge weights. The largest clique tree is very similar to the maximal clique tree and covers the important events and pathways.

## Discussion and conclusion

We introduced and analysed ten variable selection methods for disease progression models. To obtain meaningful information about the disease process, it is important to distinguish between events that significantly contribute to disease progression and events that only represent random noise. So far, only two variable selection methods were used in the literature, both based on a frequency approach. We extended this range and also considered methods that are based on pairwise interactions, on the tree model itself, and on the identification of cliques of events.

In an extensive simulation study we first optimised each method individually by finding the best parameter setting. Then, we compared all ten methods in many different data situations. It turned out that variable selection based on clique methods is very promising. Events that occur together in a certain fraction of observations are connected by an edge. In the resulting graph, we look for largest or maximal cliques and select the events associated with this clique. Only these clique methods were consistently among the best methods.

The results of this simulation study do not change if we run them again with different random seeds. We also did not include noise in the data generating process. That means the observations drawn from the underlying true tree (see step (2) of the simulation study) are all without measurement errors. Still, if such noise is included, the results are similar, as one can see in Additional file 1: Figures A.9 and A.10. We simulated the measurement errors by changing each entry of the true data matrix with probability 0.01 respectively 0.10.

Concerning our variable selection method ’independence in trees’ one might object that this method indicates that single independent events are not necessary for modelling disease progression. This is not true. We still included this method to investigate the influence of these independent events. In fact, there are about 38*%* of the true trees that possess at least one independent event in step (1) of our simulation study. Thus, sometimes this method will definitely fail in identifying all true events. Nevertheless, we wanted to analyse how this method competed against the others, and the performance was poor.

The quality of the clique methods was confirmed by the application to real data sets. Starting with two quite small but well studied data sets (meningioma and HIV) we could compare the outcome of our methods to results from the literature: The clique methods were consistent with already known facts. This was true for some other methods as well. But in the presence of additional noise variables in the meningioma data, the clique methods were the only robust ones.

To illustrate the comparability between the simulated and the real data sets, we investigate the distribution of event probabilities. Table 5 shows a summary of the occurrence rates for the events in our analysis.

**Table 5:** Overview of the occurrence rates for all events for simulated data and the three data sets

Data set	Minimum	1st quartile	Medium	Mean	3rd quartile	Maximum
simulation data	0.00	0.14	0.29	0.33	0.49	0.96
simulation data with noise	0.00	0.12	0.23	0.26	0.36	0.96
meningioma	0.02	0.07	0.04	0.08	0.06	0.38
HIV	0.12	0.20	0.24	0.27	0.36	0.42
glioblastoma	0.00	0.00	0.04	0.12	0.14	0.85

Most data sets we considered contained only few events. In the simulations we did not use more than 19 events, due to runtime constraints when calculating the induced probability distribution. However, we need methods that are also robust for larger number of events, for example when considering chromosome bands or even single genes. The analysis of the glioblastoma data set with 132 events confirmed the advantage of our clique methods in larger data sets. Almost all other variable selection methods chose too many events to fit a meaningful tree. Also when limiting the number of events to 11 only the frequency and the clique methods detected almost all events that were already mentioned in the literature.

Thus, freq and cliq are the two competitive variable selection methods. Since frequency selection is standard in the literature so far, it is no surprise that this method identifies the already known events. Nevertheless, only taking the frequency into account is not enough to cover all important events concerning disease progression. We can see this by looking at the other selected events in the glioblastoma data set. The frequency method selects additional events that were independent and did not contribute to existing pathways. However, the clique method mcliq even omits the one event known from the literature that is independent of all other events, and chooses only additional events that extend the known disease process. Thus, if we use the clique methods, which also take variable interaction into account, we can find both the important frequent events and those related to disease pathways.

We analysed these variable selection methods for the basic and popular model class of oncogenetic trees. We are aware that these models cannot represent every possible combination of events and that there can be certain observations that do not fit to the estimated model, see Table 6. Nevertheless, we believe that variable selection methods should first be investigated for a basic model class to understand their fundamental properties. As a next step, this analysis should be extended to further complex model classes, of course.

**Table 6:** Proportion of events from the three data sets that fit to the estimated model

Data set	All events	Frequency tree	Clique tree
meningioma	0.90	1.00	0.97
HIV	0.87	0.94	0.88
glioblastoma	0.79	0.69	0.59

For the glioblastoma data the numbers are lower due to the tree depth of 4 and 6 for freq and cliq, respectively. For the simulated data, minimum, 1st quartile, median, mean, 3rd quartile and maximum are 0.47, 0.94, 0.99, 0.92, 1.00 and 1.00

## Additional file

Additional file 1
**Figure A.1.** Illustrating example concerning the difference between largest and maximal cliques. **Figure A.2.** Cluster dendrogram of the L1-distances using the complete linkage approach to potentially restrict the number of parameter combinations. **Figure A.3.** Results of the univariate frequency method for the *L*_2_-distances respectively cosine-distances. **Figure A.4.** Results of the missing six variable selection methods. Based on these graphics one can identify the best threshold. **Figure A.5.** Results of the simulation study for the two criteria sens and spec where *α*_*l*_=0.2. **Figures A.6** and **A.7.** Results of sens vs. spec for 16 different data situations. **Figure A.8.** Remaining trees resulting from the variable selection process concerning the glioblastoma data set. **Figure A.9.** Scatterplots of true data vs. contaminated data. **Figure A.10.** Scatterplots of true data vs. data with 10% noise. **Table B.1.** List of the 32 parameter settings representing the different data situations that are investigated by our variable selection methods. **Table B.2.** List of events from the extended meningioma data set (39 additional variables with a random frequency of 0.5%) that were chosen by our variable selection methods using the thresholds from the simulation study. (PDF 409 kb)
