# Supplementary material for: Variable selection for disease progression models: methods for oncogenetic trees and application to cancer and HIV
Source: BMC Bioinformatics. 2017 Aug 1;18:358. doi: 10.1186/s12859-017-1762-1 (PMC5539896; doi:10.1186/s12859-017-1762-1)
Supplement: Additional file 1 — Figure A.1. Illustrating example concerning the difference between largest and maximal cliques. Figure A.2. Cluster dendrogram of the L1-distances using the complete linkage approach to potentially restrict the number of parameter combinations. Figure A.3. Results of the univariate frequency method for the L 2-distances respectively cosine-distances. Figure A.4. Results of the missing six variable selection methods. Based on these graphics one can identify the best threshold. Figure A.5. Results of the simulation study for the two criteria sens and spec where α l=0.2. Figures A.6 and A.7. Results of sens vs. spec for 16 different data situations. Figure A.8. Remaining trees resulting from the variable selection process concerning the glioblastoma data set. Figure A.9. Scatterplots of true data vs. contaminated data. Figure A.10. Scatterplots of true data vs. data with 10% noise. Table B.1. List of the 32 parameter settings representing the different data situations that are investigated by our variable selection methods. Table B.2. List of events from the extended meningioma data set (39 additional variables with a random frequency of 0.5%) that were chosen by our variable selection methods using the thresholds from the simulation study. (PDF 409 kb) [file 12859_2017_1762_MOESM1_ESM.pdf]

Additional file for "Variable Selection for Disease Progression Models: Methods for Oncogenetic Trees and Application to Cancer and HIV"

Katrin Hainke Sebastian Szugat Roland Fried Jörg Rahnenführer

Fakultät Statistik, TU Dortmund, 44221 Dortmund, Germany

{hainke, szugat, fried, rahnenfuehrer}@statistik.tu-dortmund.de

A. Additional Figures

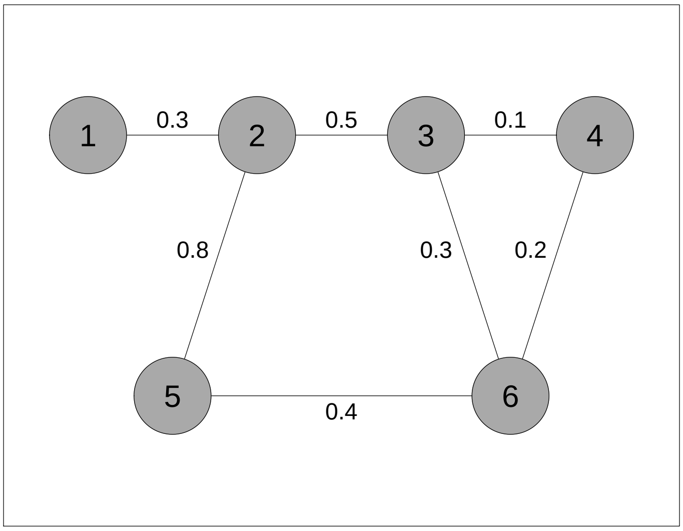

Figure A.1: Illustrating example concerning the difference between largest and maximal cliques. A *clique* is a subgraph, where all vertices are connected by an edge. In this example, we have 8 different cliques: 1-2, 2-3, 2-5, 3-4, 3-6, 4-6, 5-6, 3-4-6. The *largest clique* is the clique with the most vertices, in this example the clique 3-4-6, because there is no other clique with more than three vertices. A *maximal clique* cannot be extended to a larger clique. In this example, we have 5 maximal cliques: 1-2, 2-3, 2-5, 5-6, 3-4-6. The clique 3-4 is no maximal clique, because including vertice 6 still results in a clique. The edge weights are not necessary for identifying largest or maximal cliques, but they are needed to find the *maximum weight clique*. This is a maximal clique with the highest sum of edge weights. In this example, the maximum weight clique is 2-5 with a weight of 0.8. The largest clique 3-4-6 only has a weight of 0.6.

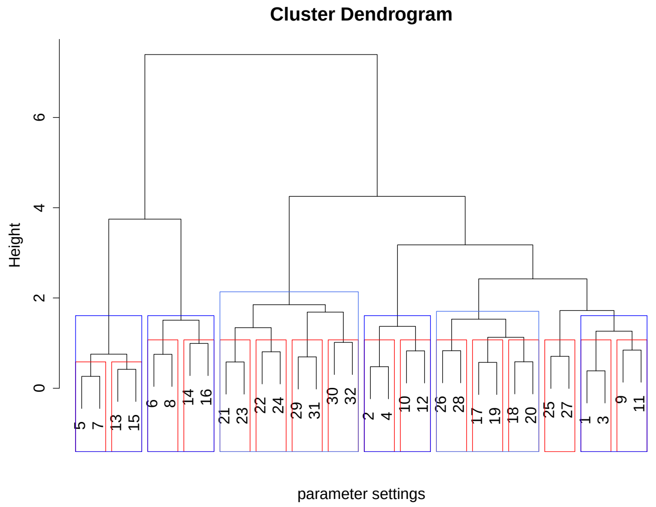

Figure A.2: Cluster dendrogram of the L_1-distances using the complete linkage approach to potentially restrict the number of parameter combinations. The parameter settings of the first conjunction (red boxes) only differ in the number n_1 of true events. Those of the second conjunction are nearly always the ones where α_l is involved (blue boxes). The 32 different parameter settings are specified in Table B.1 an page 11.

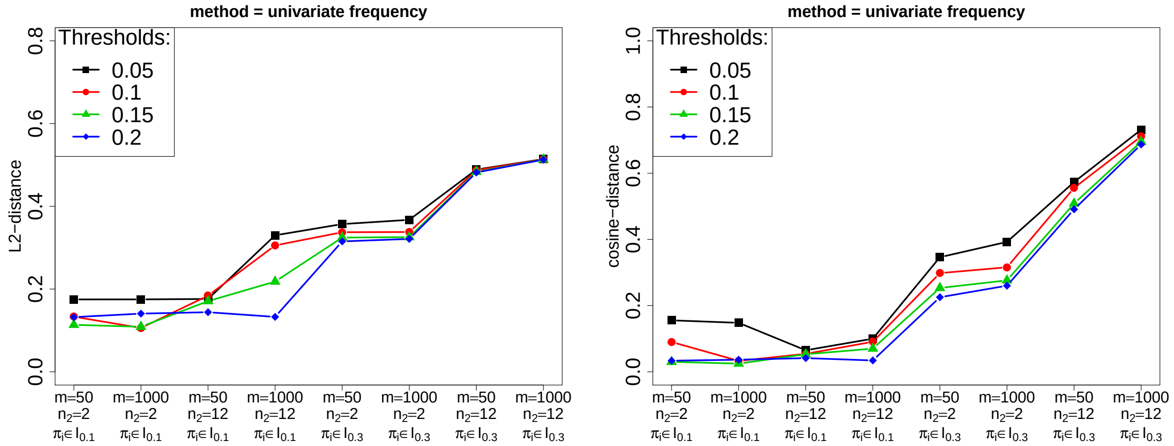

Figure A.3: Results of the univariate frequency method. In comparison to the L_1-distances (top left of Figure 2 in the paper) one can now see the means of the 100 L_2-distances (left) respectively cosine-distances (right).

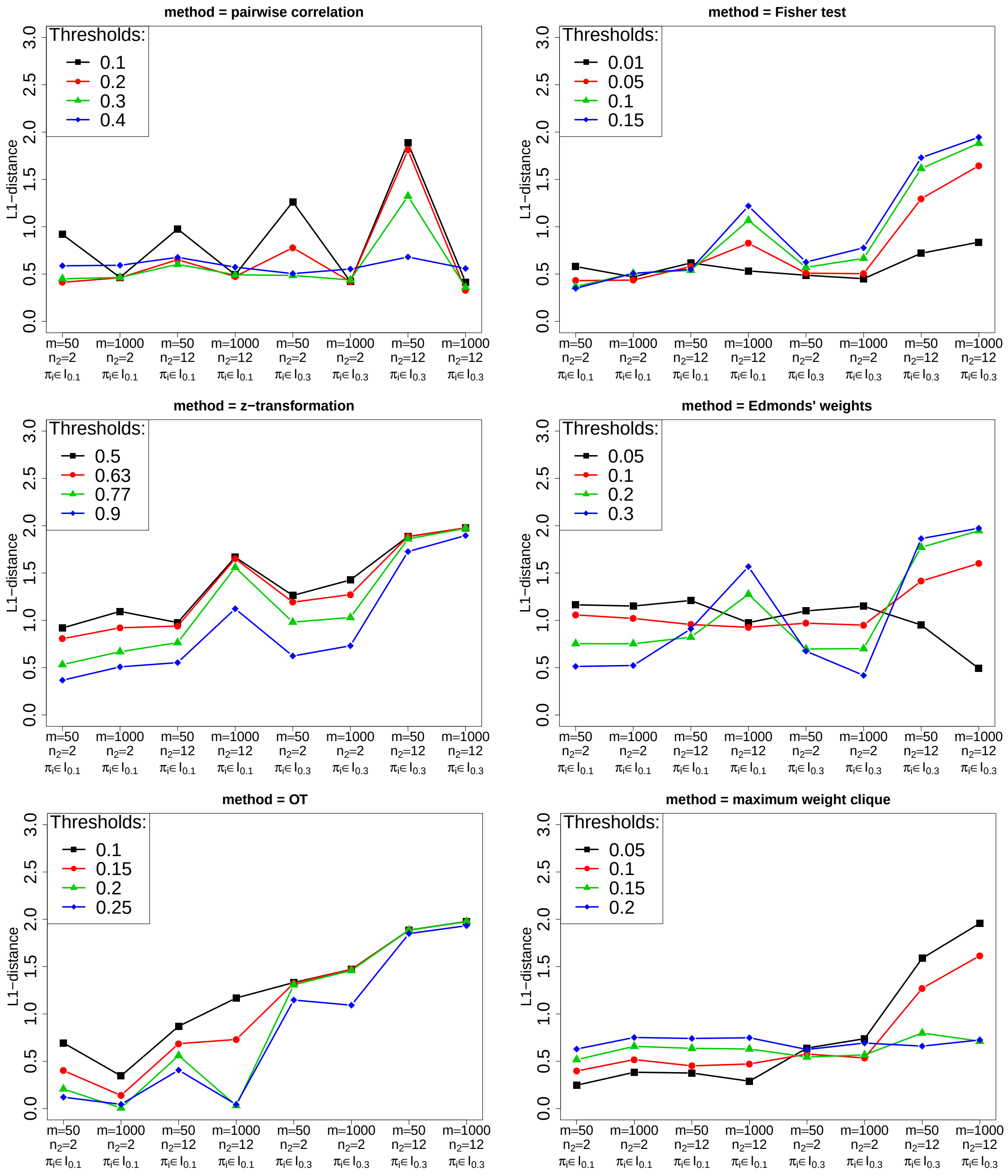

Figure A.4: Results of the missing six variable selection methods. Based on these graphics one can identify the best threshold.

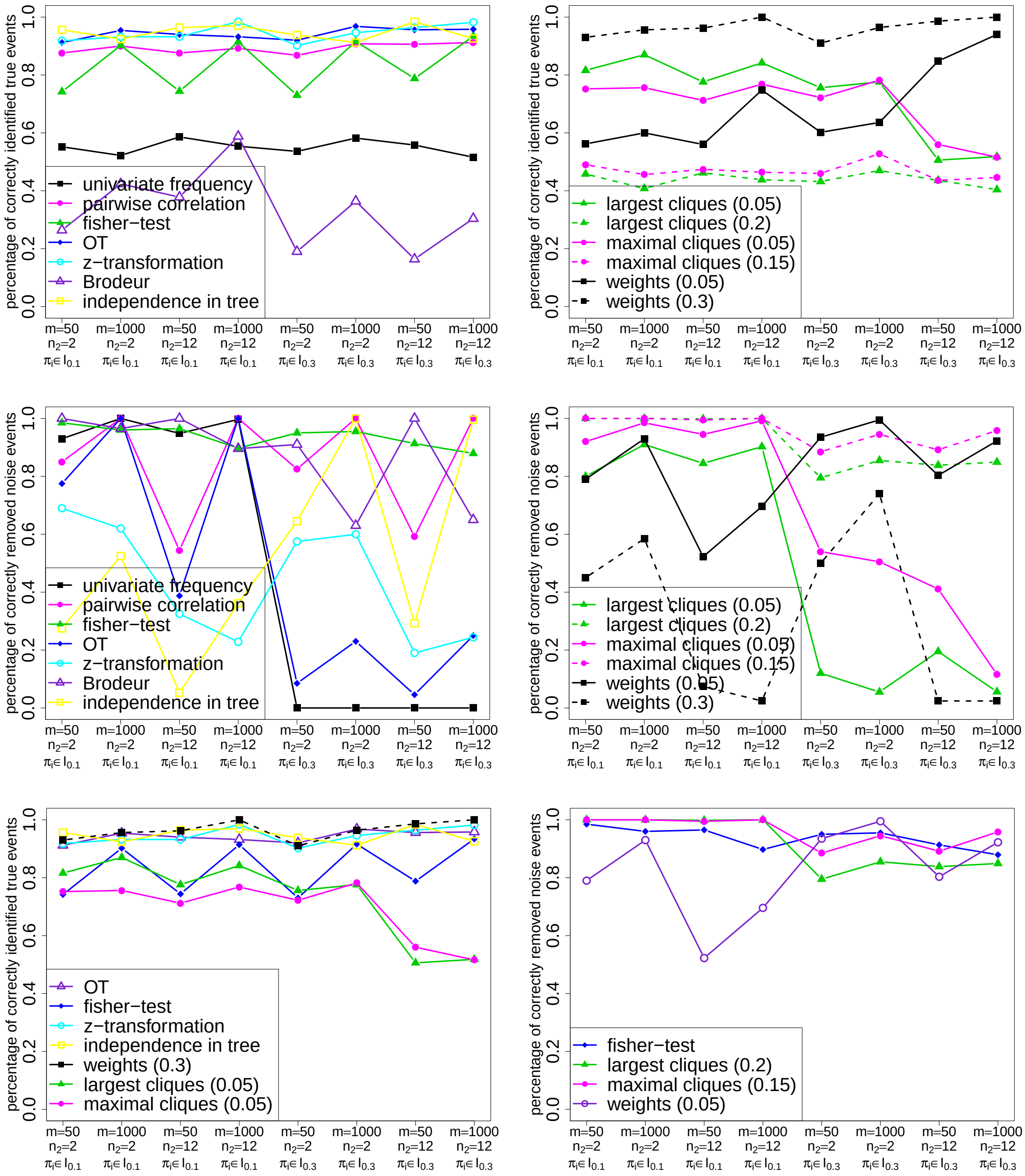

Figure A.5: Results of the simulation study. The eight different parameter settings are displayed on the x-axis whereas the means of the 100 values for `sens` and `spec` are shown on the y-axis. *Top row:* Results for the criterion `sens`, left: comparison of all seven methods with one overall best threshold, right: comparison of all three methods with two thresholds depending on the underlying data situation. *Middle row:* Results for the criterion `spec`, left: comparison of all seven methods with one overall best threshold, right: comparison of all three methods with two thresholds depending on the underlying data situation. *Bottom row:* Comparison of all variable selection methods for the two criteria `sens` (left) and `spec` (right).

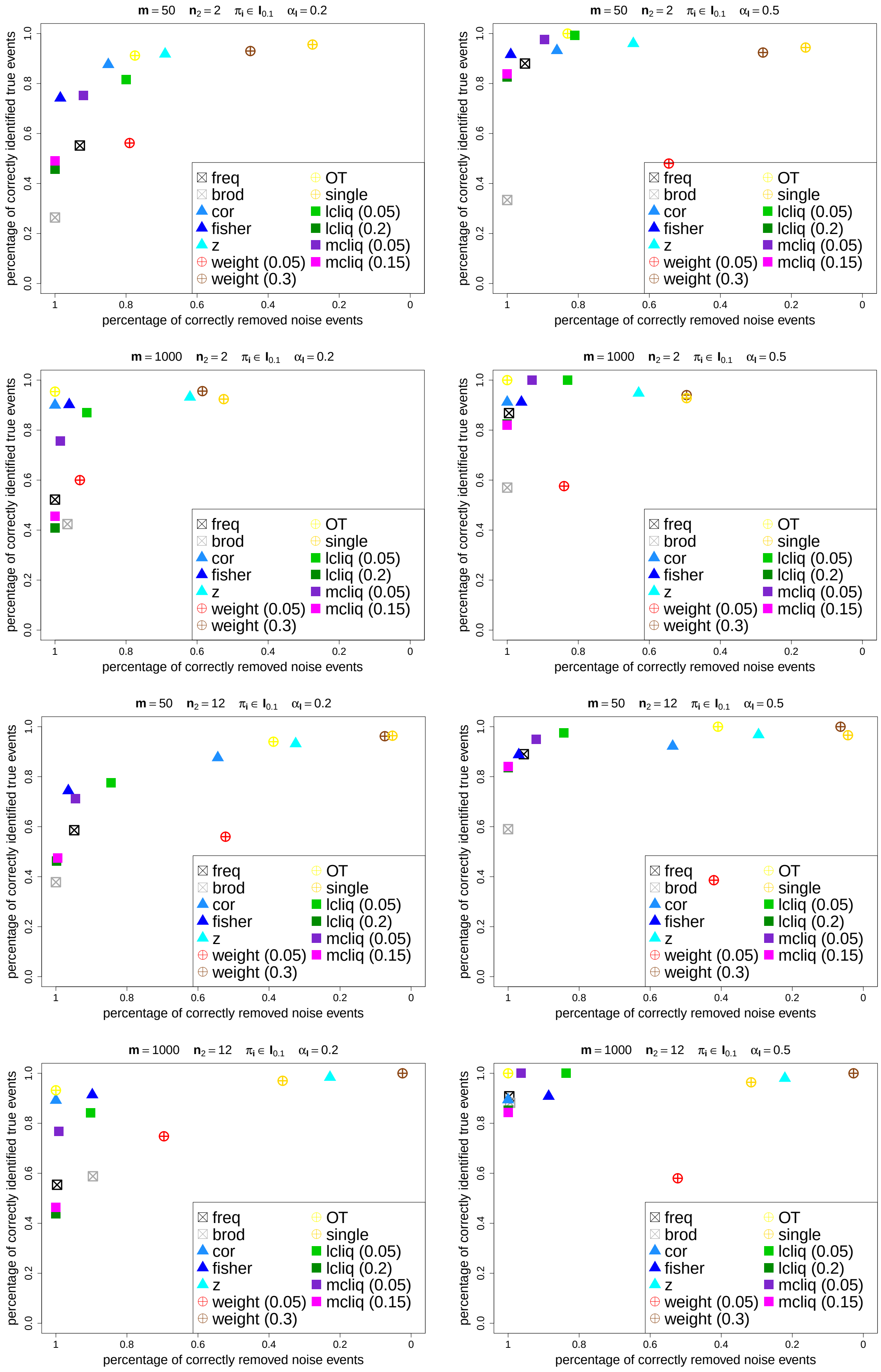

Figure A.6: For explanation, see page 7.

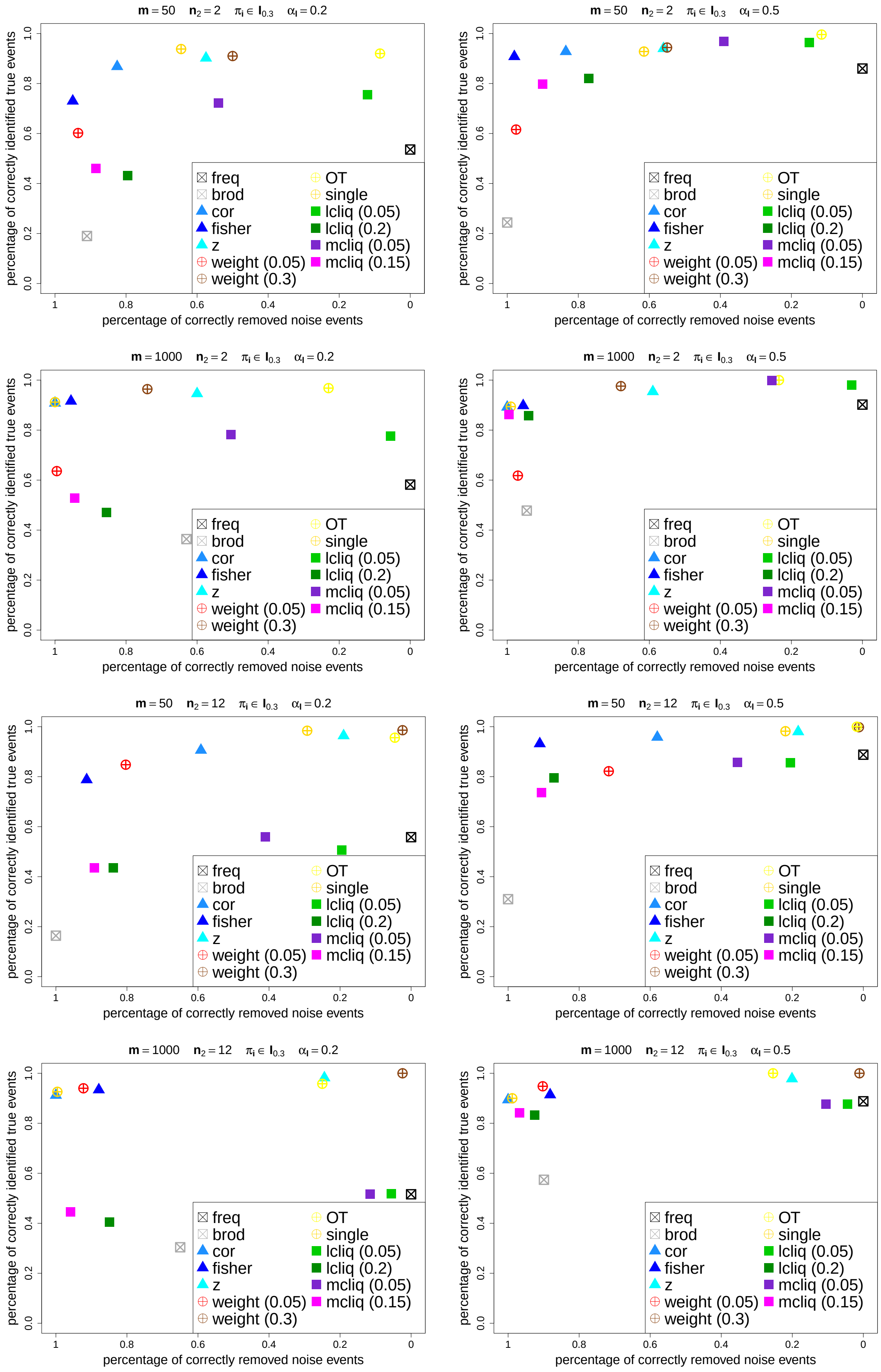

Figure A.7: For explanation, see page 7.

Explanation for Figures A.6 and A.7:

The 16 panels in Figures A.6 and A.7 show 16 different data situations. For the ones on the left hand side it holds α_I = 0.2, and for the ones on the right α_I = 0.5. The order of the data situations per row equals the one we always used in other graphics for the x-axis. In these graphics, we simultaneously show the results of the criteria `sens` and `spec`, similar to a ROC-curve. The percentages of correctly identified true events (on a scale from 1 to 0) are shown on the x-axis and the percentages of correctly removed noise events on the y-axis. Each method is represented by a certain coloured symbol. A variable selection method achieves good results, if it lies in the upper left corner, i.e. if it identifies most of the true events and removes most of the noise events. A method can be regarded as overall good, if it lies in the upper left corner for all different data situations.

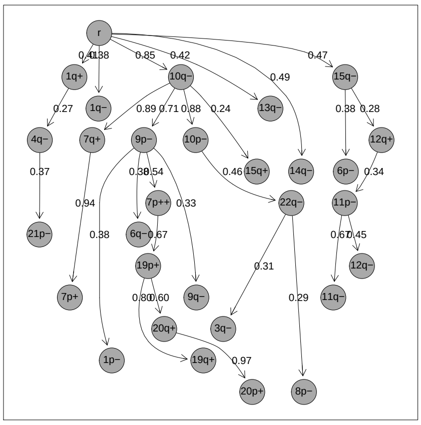

(a) Brodeur tree

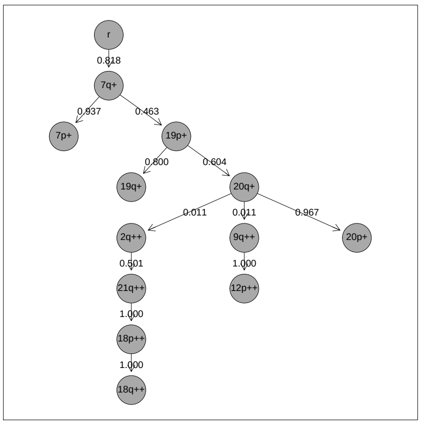

(b) correlation tree

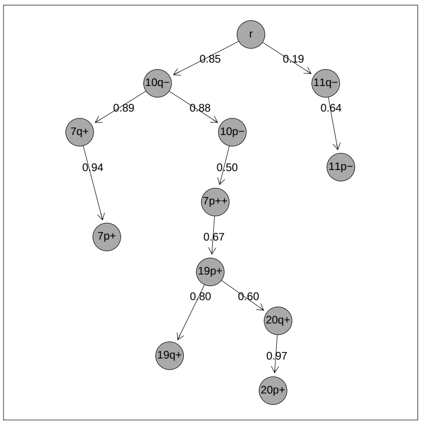

(c) fisher test tree

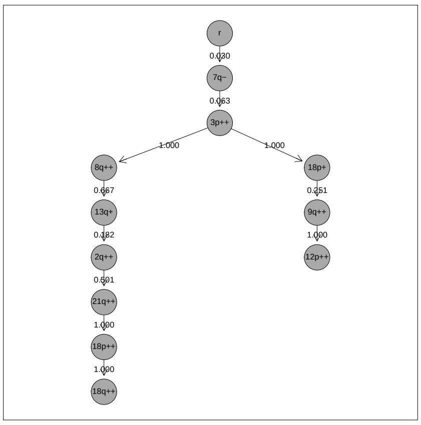

(d) weight tree

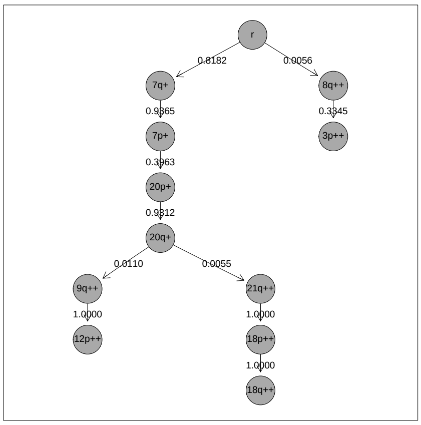

(e) OT tree

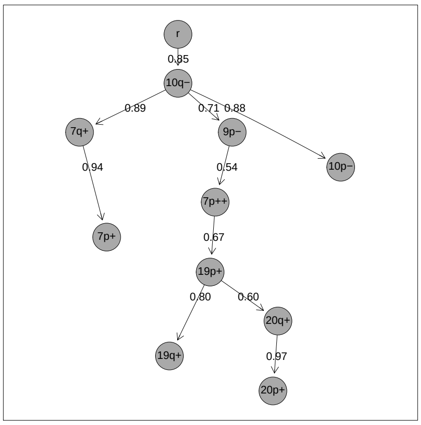

(f) largest cliques tree

Figure A.8: Remaining trees resulting from the variable selection process concerning the glioblastoma data set.

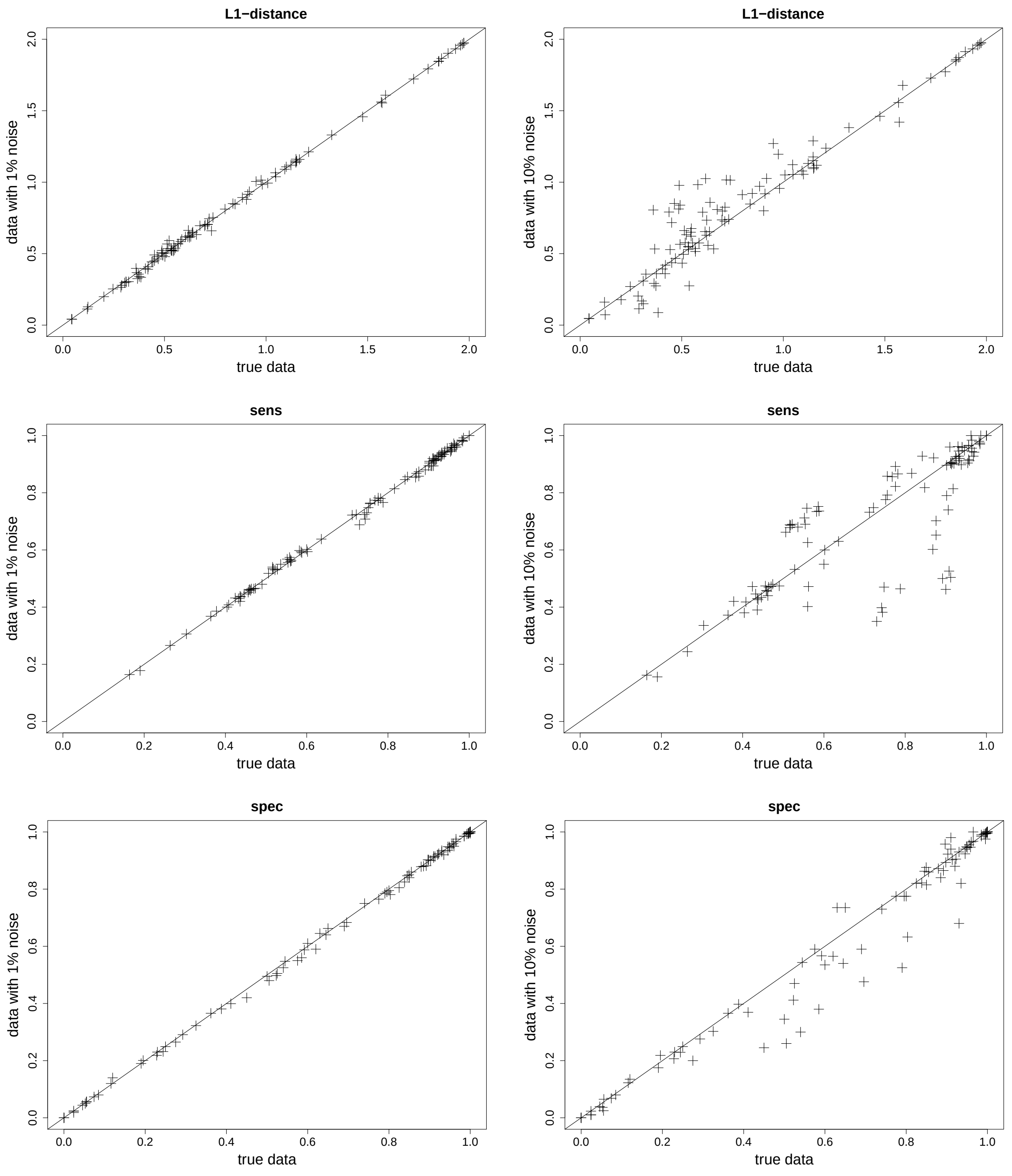

Figure A.9: Scatterplots of true data versus contaminated data to show the influence of noise in the data generating process. For all 10 methods, each with their best threshold(s), and all 3 distance measures we show how the results differ if the data drawn from the true model suffers from some measurement errors. We simulate these measurement errors by changing each entry of the true data matrix with probability 0.01 respectively 0.10. If this noisy data has no influence on the results, the points of the scatterplot will all be on the diagonal.
We see that this holds for all figures on the left hand side (1% noise). If there is more noise in the data, the results differ a bit but not severely. In general, the interpretation of the results and the ranking of methods stay the same. An explanation why sometimes the results are better if there is noise in the data is given in Figure A.10.

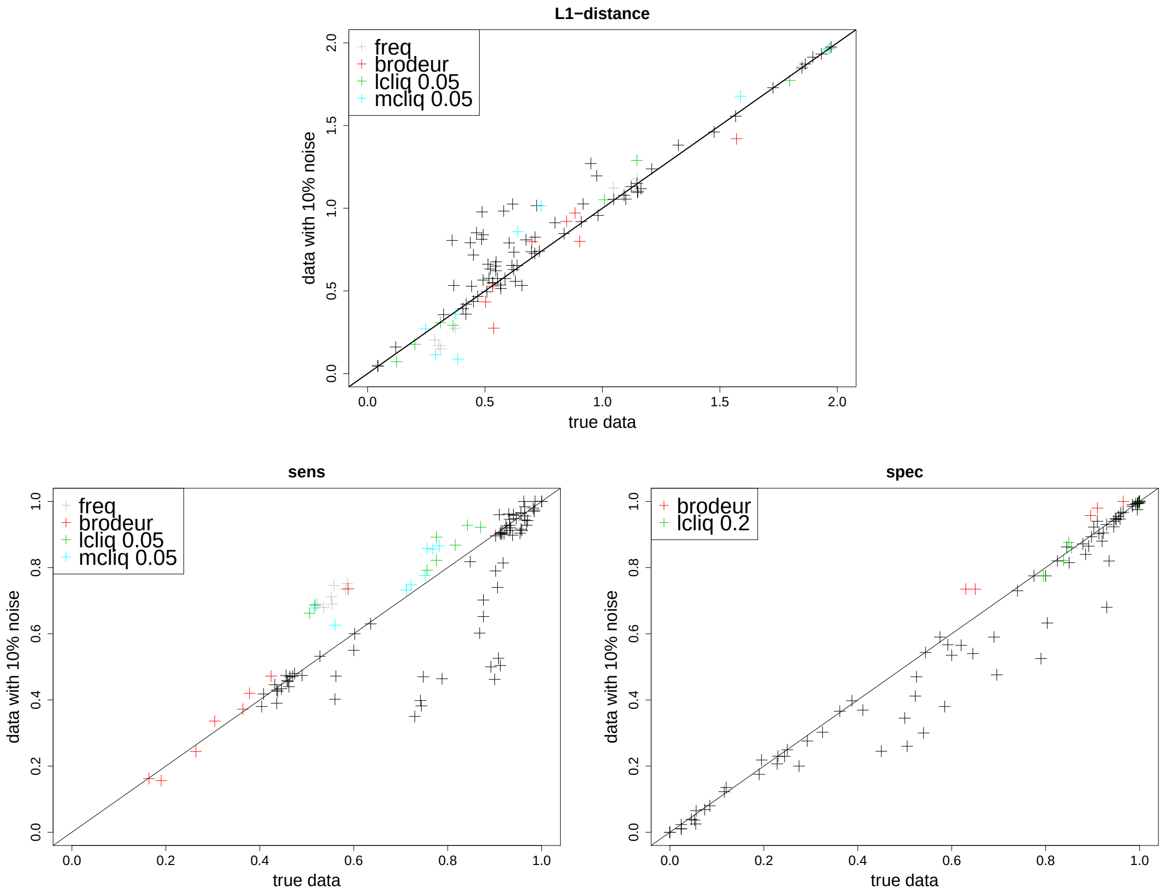

Figure A.10: The same scatterplots as in Figure A.9 (right hand side), but now with some colouring to explain why sometimes the results for the noisy data are better than the ones for the true data. Note that results for the L_1-distance are better with noisy data if they lie under the diagonal, whereas for the other two distance measures they lie above. Largest differences are observed for the frequency and Brodeur method as well as the two clique methods.
Some of the 5 true events may have little probability due to their late appearance in the true model. If we now change every entry in the data matrix with probability 0.10 it is more likely for those rare events to change 0s to 1s than vice versa. Thus, these events gain an overall higher probability. For the frequency method this results in a better outcome in detecting more of the 5 true events, because the frequency of those true events might now exceed the chosen threshold. Concerning the Brodeur method, having a higher frequency for true events results in a higher threshold, such that more noise events are neglected. The higher probability for the true events is also beneficial for the clique methods since only events which have been observed simultaneously with at least one other event can be selected. If their overall probability of appearance is already below the chosen threshold, they get no chance of being selected at all.

B. Additional Tables

Table B.1: List of the 32 parameter settings representing the different data situations that are investigated by our variable selection methods. ($I_{0.1}$ = [0, 0.2], $I_{0.3}$ = [0.2, 0.4])

	m	n_1	n_2	π_i	α_l
1	50	5	12	$I_{0.1}$	0.2
2	1000	5	12	$I_{0.1}$	0.2
3	50	7	12	$I_{0.1}$	0.2
4	1000	7	12	$I_{0.1}$	0.2
5	50	5	12	$I_{0.3}$	0.2
6	1000	5	12	$I_{0.3}$	0.2
7	50	7	12	$I_{0.3}$	0.2
8	1000	7	12	$I_{0.3}$	0.2
9	50	5	12	$I_{0.1}$	0.5
10	1000	5	12	$I_{0.1}$	0.5
11	50	7	12	$I_{0.1}$	0.5
12	1000	7	12	$I_{0.1}$	0.5
13	50	5	12	$I_{0.3}$	0.5
14	1000	5	12	$I_{0.3}$	0.5
15	50	7	12	$I_{0.3}$	0.5
16	1000	7	12	$I_{0.3}$	0.5
17	50	5	2	$I_{0.1}$	0.2
18	1000	5	2	$I_{0.1}$	0.2
19	50	7	2	$I_{0.1}$	0.2
20	1000	7	2	$I_{0.1}$	0.2
21	50	5	2	$I_{0.3}$	0.2
22	1000	5	2	$I_{0.3}$	0.2
23	50	7	2	$I_{0.3}$	0.2
24	1000	7	2	$I_{0.3}$	0.2
25	50	5	2	$I_{0.1}$	0.5
26	1000	5	2	$I_{0.1}$	0.5
27	50	7	2	$I_{0.1}$	0.5
28	1000	7	2	$I_{0.1}$	0.5
29	50	5	2	$I_{0.3}$	0.5
30	1000	5	2	$I_{0.3}$	0.5
31	50	7	2	$I_{0.3}$	0.5
32	1000	7	2	$I_{0.3}$	0.5

Table B.2: List of events from the extended meningioma data set (39 additional variables with a random frequency of 0.5%) that were chosen by our variable selection methods using the thresholds from the simulation study (x = event was selected). The largest and maximal cliques with thresholds 0.2 and 0.15 respectively selected no events at all. The row labelled with '# random' gives the number of selected variables out of the 39 additional ones. The last two rows specify the threshold, one needs to choose to select all of the 9 'true' variables and as little of the random ones as possible as well as the number of selected noise variables ('-1' means that one 'true' variable could not be selected).

method	`freq`	`brod`	`cor`	`fisher`	`z`	`weight`	`weight`	`OT`	`single`	`lcliq`	`mcliq`
threshold	0.2	0.04	0.3	0.01	0.9	0.05	0.3	0.25	-	0.05	0.05
Chr14-		x	x	x	x	x	x	x	x	x	x
Chr22-	x	x	x	x	x	x	x	x	x	x	x
Chr1p-		x	x	x	x	x	x	x	x	x	
Chr6-			x	x	x	x	x	x	x		
Chr10-			x	x	x	x	x		x		
Chr18-			x	x	x	x	x	x	x		
Chr19-			x	x	x	x	x		x		
ChrY-		x		x	x	x	x	x	x		
ChrX-		x		x	x	x	x		x		
# random	0	0	8	6	20	23	28	8	36	0	0
'optimal' τ	0.0166	-	0.1940	0.0015	0.9999999	0.0480	-	0.1801	-	0.0045	0.0030
# random	0	-	11	2	11	21	-	12	-	0	-1
